# A six degrees-of-freedom cable-driven robotic platform for head–neck movement

**DOI:** 10.1038/s41598-024-59349-0

**Published:** 2024-04-16

**Authors:** Ian Bales, Haohan Zhang

**Affiliations:** Robotics Center and Department of Mechanical Engineering, University of Utah, Salt Lake City, UT 84112 USA

**Keywords:** Mechanical engineering, Biomedical engineering

## Abstract

This paper introduces a novel cable-driven robotic platform that enables six degrees-of-freedom (DoF) natural head–neck movements. Poor postural control of the head–neck can be a debilitating symptom of neurological disorders such as amyotrophic lateral sclerosis and cerebral palsy. Current treatments using static neck collars are inadequate, and there is a need to develop new devices to empower movements and facilitate physical rehabilitation of the head–neck. State-of-the-art neck exoskeletons using lower DoF mechanisms with rigid linkages are limited by their hard motion constraints imposed on head–neck movements. By contrast, the cable-driven robot presented in this paper does not constrain motion and enables wide-range, 6-DoF control of the head–neck. We present the mechatronic design, validation, and control implementations of this robot, as well as a human experiment to demonstrate a potential use case of this versatile robot for rehabilitation. Participants were engaged in a target reaching task while the robot applied both assistive and resistive moments on the head during the task. Our results show that neck muscle activation increased by 19% when moving the head against resistance and decreased by 28–43% when assisted by the robot. Overall, these results provide a scientific justification for further research in enabling movement and identifying personalized rehabilitation for motor training. Beyond rehabilitation, other applications such as applying force perturbations on the head to study sensory integration and applying traction to achieve pain relief may benefit from the innovation of this robotic platform which is capable of applying controlled 6-DoF forces/moments on the head.

## Introduction

The head is connected to the trunk through a highly flexible cervical spine that consists of multiple cervical joints. The cervical spine allows the head to have six degrees-of-freedom (DoF) mobility relative to the trunk^1,2^. While most daily activities require head rotations, translational movements of the head are observed^3,4^. The mobility of the head is imperative to human survival^5^; however, various neurological disorders can lead to impaired head–neck movements. For example, neck muscle weakness caused by diseases like amyotrophic lateral sclerosis (ALS) and cerebral palsy (CP) can result in dropped head posture and/or poor motor control of the head^6–8^. Static neck collars are used in clinical practices to keep the head upright^9,10^, but they are uncomfortable and restrictive^11^. More importantly, these static collars fail to restore head–neck movement, which leads to atrophied muscles and challenges with engaging in social interactions (e.g., making eye contact)^12^. Stabilization exercises are also common to strengthen and improve control of neck muscles, but these exercises lack control and repeatability when applied manually^13,14^. Therefore, there is a clinical need to develop solutions for these head–neck rehabilitative applications.

Powered robotic neck devices have been developed to address this unmet clinical need. However, most systems focus only on a subset of the DoF of the head. For example, a recent powered neck exoskeleton with 3-DoF was developed to enable large head rotations while coupling the head translations with rotations through an optimization based on empirical movement data from human subjects^4^. Although this approach has demonstrated that it can enable head–neck rotations for multiple applications^15–20^, the device can be restrictive to natural head–neck movements of different users due to the constraints imposed by the linkage kinematics which couples the rotations and translations of the head. By contrast, cable-driven designs have the potential to address the issue caused by rigid kinematics as cables apply tensile forces to collectively generate force and moment on the head without imposing hard kinematic constraints^21^. Recently, there have been efforts to incorporate cables in head–neck device designs^22,23^. However, these existing devices are unable to fully control the 6-DoF movement and force/moment application on the head–neck, which limits their potential for clinical and scientific applications. Additionally, rigid linkages are often still used, which restrict natural head–neck movement during use. Importantly, fundamental questions like how to design and control cable-driven robots for 6-DoF natural head–neck movements and how human users respond to the interaction with the cable-driven neck device remain unanswered.

To fill the research gap, we recently developed a novel cable-driven robotic platform for 6-DoF head–neck movements in the Utah Wearable Robotics Laboratory at the University of Utah. Our long-term goal is to leverage the versatility of this system to study how to provide motion assistance and train head control using cable-driven designs. In this paper, we introduce its design and control principles, benchtop validation studies of its 6-DoF control capabilities, as well as a case study in healthy human participants to demonstrate its potential use for rehabilitation. Our results show that this robotic platform can accurately quantify the 6-DoF head–neck motions and control the 6-DoF force and moment applied on the head. We also show that in healthy participants, neck muscle activation can be modulated in a controlled manner, measured by electromyography (EMG), using resistive and assistive controllers that apply 3-DoF moments on the head. Here, our main contributions are: (1) innovation of a powered head–neck system that allows for 6-DoF natural head–neck motions and quantification of its 6-DoF measurement and force/moment control capabilities, and (2) a biomechanical characterization of human response (kinematics and muscle activation) during interaction with this head–neck device when controlled moments are applied on the head.

Results acquired from this study can help inform future development of new design and control paradigms to answer fundamental research questions and to translate robotic head–neck systems for use outside the laboratory setting. Beyond rehabilitation, there is an array of other scientific and clinical applications which can potentially benefit from this innovation. For example, as the head houses multiple important sensory organs such as vestibular and visual systems, there is growing interest in studying how human participants integrate and process these sensory inputs through techniques like applying directional force perturbations on the head^24–26^. Additionally, cervical deformity surgery and other spinal maneuvers require force and moment to be applied on the head to help achieve optimal cervical joint alignment and pain relief^27^. Because the proposed robotic system allows for full 6-DoF head–neck motion and force/moment application on the head, it can potentially be used in these applications with small design and control modifications.

## Mechatronic design

In this section, we first provide the necessary kinematics background of a general cable-driven parallel mechanism, followed by the description of design of a chair-mounted, easy-to-reconfigure physical prototype. Finally, we introduce control modes implemented in this robot for its potential applications.

**Figure 1:**
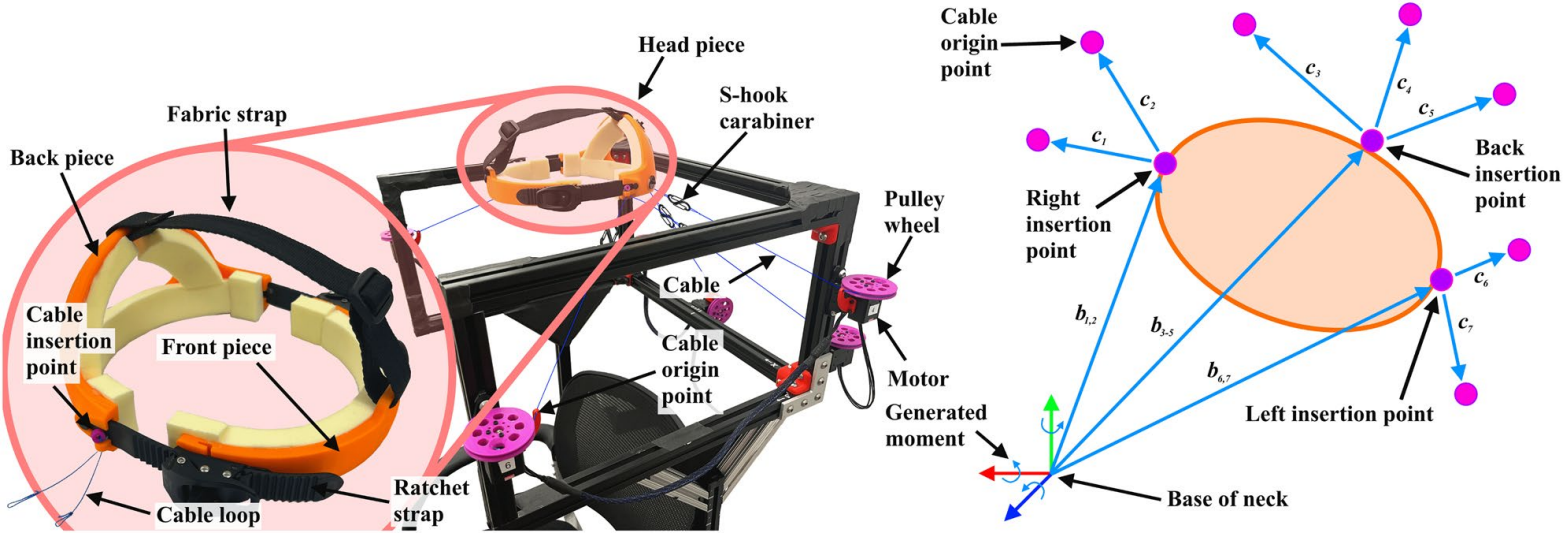
Physical robot and kinematics design. Left: Chair mounted robot. The size of the frame is 51 $\times$ 46 $\times$ 28 cm. The height of the device is adjustable, fitting users between 165–185 cm tall. Seven servo motors (Dynamixel XM430-W210-R, ROBOTIS, Seoul, South Korea) are mounted to the frame. Mounted to each motor is a spool with radius $\rho =25$ mm, as well as a fixed cable guide to constrain the positions of the *cable origin points*. Nylon braided cables are wrapped around each spool. An s-hook carabiner is attached to the other end of each cable for attachment to the head piece. Inset: Head piece. Two 3D-printed pieces fit around the the front and back of the head. The two pieces are connected by rigid ratchet straps on either side of the head. The ratchet system allows for fine size adjustment to comfortably fit a range of head circumferences from 47–59 cm while maintaining structural rigidity. An adjustable fabric strap keeps the head piece in place, counteracting the downward pull of the cables. At three different *cable insertion points*, seven short cable loops are affixed to attach to the s-hook carabiners. Right: Kinematic overview of the robot. The global origin and center of rotation is located at the base of the neck at approximately the C7 vertebra. All commanded moments are applied about this location.

### Kinematics and physical design

A general cable-driven parallel mechanism consists of multiple cables attached to the robot end-effector. When the cables are tightened, collectively, a wrench (force and moment) is applied on the end-effector. Because cables can only transmit tensile loads and not compressive ones, redundancy is necessary in a cable-driven robot; at least one more cable than the number of controlled degrees of freedom is required. We choose to use seven cables in our design to allow for full control of the 6-DoF movement of the head–neck. Assuming quasi-static behavior, a 6-DoF wrench ${\textbf{w}}$ applied on the end-effector is related to the 7 cable tensions ${\textbf{t}}$ by

1 $$\begin{aligned} {\textbf{w}}=A{\textbf{t}}, \end{aligned}$$

where $${\textbf{w}}=\begin{bmatrix}{\textbf{f}}^\text {T}&{\textbf{m}}^\text {T}\end{bmatrix}^\text {T}$$, ${\textbf{f}}$ and ${\textbf{m}}$ are $3\times 1$ force and moment vectors, respectively, and $${\textbf{t}}=\begin{bmatrix}t_1&t_2&\cdots&t_7\end{bmatrix}^\text {T}$$. Matrix *A* is a $6\times 7$ wrench matrix that depends on the configuration of the robot:

2 $$\begin{aligned} A= \begin{bmatrix} \hat{{\textbf{c}}_1} &{} \cdots &{} \hat{{\textbf{c}}_i} &{} \cdots &{} \hat{{\textbf{c}}_7}\\ {\textbf{b}}_1\times \hat{{\textbf{c}}_1} &{} \cdots &{} {\textbf{b}}_i\times \hat{{\textbf{c}}_i} &{} \cdots &{} {\textbf{b}}_7\times \hat{{\textbf{c}}_7} \end{bmatrix}, \end{aligned}$$

where ${\textbf{b}}_i$ is the position vector from the base of the neck to each cable’s insertion point, and $\hat{{\textbf{c}}_i}$ is the unit vector pointing from each cable’s insertion point to the origin. Specifics of the control and forward kinematics of this robot are detailed in the low-level control section. Each cable is actuated by a servo motor mounted on the edges of a cuboid frame (Fig. 1, left). This frame is then mounted on a chair so that a user can be seated while using this device. The mounting positions of these motors can be easily adjusted on the frame based on different design criteria.

Each cable originates from a spool connected to the shaft of a servo motor and attaches to the end-effector, or head piece (Fig. 1, inset). The head piece would be rigidly attached to the head of the user, allowing the cables to transmit a wrench to the user’s head. The rigidity of the head piece improves comfort as it distributes the point-loading from the cables evenly around the head of the wearer. In the current design, we chose to place two cables on either side of the user and the remaining three cables on the back of the user to maintain a symmetric design and to not impede upon the user’s visual field. The cables on the same side (i.e., left, right, and back) intersect at a point on the respective side of the end-effector, resulting in a total of three attachment points on the head piece. With this configuration, a geometric solution exists to solve for the position and orientation of the head piece based on the cable lengths measured by the servo motors (i.e., forward kinematics)^28^. Because the cable structure only applies forces and moments but does not impose constrained kinematics, this device can accommodate wide-range, natural movement of the head–neck. Key parameters defining the kinematics of the robot are presented in Fig. 1, Right. In this paper, flexion-extension is positive when extending the head backward about the x-axis, lateral bending is positive when bending to the right about the y-axis, and twist corresponds to axial rotation of the neck and is positive when turning to the left about the z-axis.

### Optimization of cable geometry

The cable attachment positions affect the transmission efficiency of the device. For this robot’s rehabilitative application, we chose to optimize these positions so that cable tensions can be most efficiently transmitted to a resultant moment applied on the head throughout its natural range of motion, that is, relatively small cable tensions are necessary to generate relatively large output moments. This optimization strategy is beneficial because smaller actuators can then be used to achieve the necessary moment output and small head movements can be more reliably measured. This strategy is specifically tailored to maximizing efficiency of head–neck movements unlike other methods^29–32^.

To achieve this outcome, we defined the following optimization problem:

3 $$\begin{aligned} \underset{{\textbf{x}}}{\arg \max }~f({\textbf{x}})=\sum _{d=1}^{3}\left( \sum _{i=1}^{7}\left| \frac{\Delta l_i}{\Delta \theta _d}\right| -\left| \sum _{i=1}^{7}\frac{\Delta l_i}{\Delta \theta _d}\right| \right) , \end{aligned}$$

where ${\textbf{x}}$ is the parameter set, and $\frac{\Delta l_i}{\Delta \theta _d}$ denotes the transmission ratio between the change of length of the $i{\text {th}}$ cable ($i=1,2,\ldots ,7$) and the change of head pose in the $d{\text {th}}$ direction ($d=1,~2,~3$). Each solution ${\textbf{x}}$ consists of eight parameters $x\in (0,1)$ that fully define the 3D positions of the cable origin and insertion points. One parameter defines the position of the right insertion point along the head piece; its height is fixed. One parameter is used to define the height of the $4{\text {th}}$ cable, which is otherwise placed in the midsagittal plane. For cables 1–3, two parameters each are used to define their 2D position in the plane that is the face of the cuboid frame the cable insertion point exists upon. The remaining cables are generated by reflecting cables 1–3 across the midsagittal plane. These parameters always produce a feasible robot design that is symmetric about the midsagittal plane where each cable originates from the device’s frame and inserts at the head piece in the aforementioned 2-3-2 pattern.

The first term of Eq. (3) ensures that we search for an optimal design that achieves a large change in cable lengths when there is a small change in head angles throughout the range of motion of the head, thereby improving the robot’s ability to measure small head movements. Based on the principle of virtual work, smaller cable tensions will result in a relatively larger output moment, thereby most efficiently transmitting the cable forces into the 3D moment applied on the head. The second term ensures that the robot is equally efficient in both directions about a given axis of rotation. This objective enables the use of a computer model of the head–neck for evaluating the performance of the robot across the natural range of motion of the head–neck.

**Figure 2:**
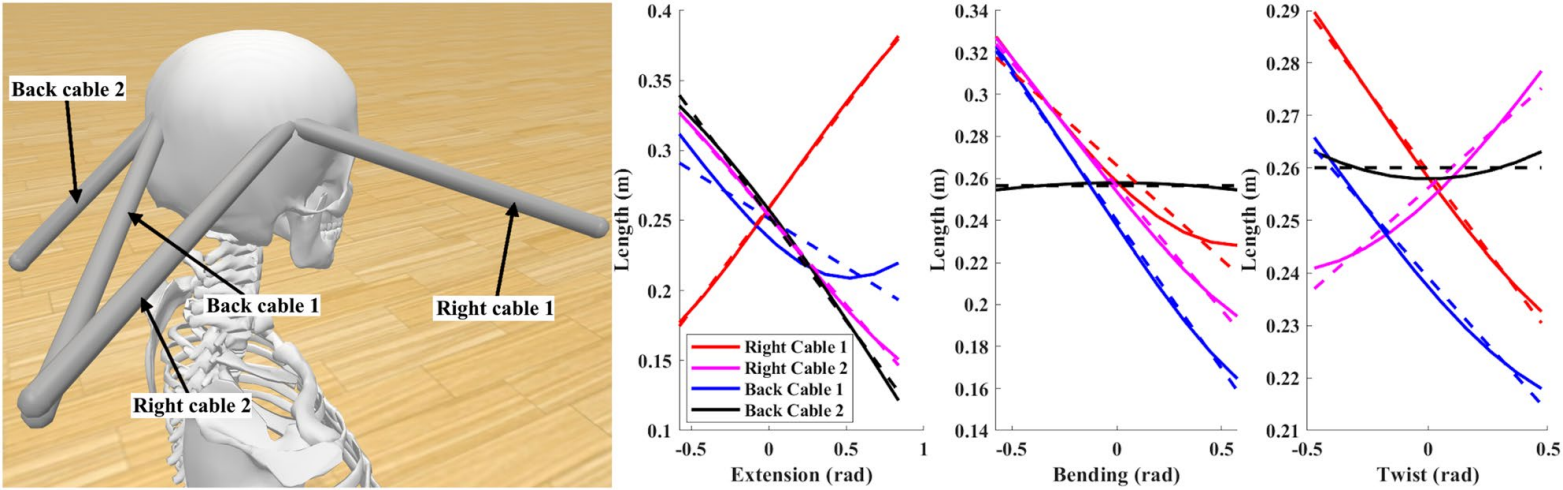
Optimization results. Left: OpenSim model of cable positions as identified in optimization. Right: Cable length vs. angle for each direction of movement. Solid lines represented simulation data; dashed lines show best-fit line. For flexion, the remaining cables have identical curves to those shown; for bending and twist, the remaining cables have curves with opposite slope to the corresponding ones shown.

A biomechanical neck model in OpenSim^33^ was used to generate realistic neck motion. The ranges of motion of this model are: $-33\text {--}48$° flexion-extension, $\pm 33$° lateral bending, and $\pm 27$° twist. The cable lengths and head angles were computed during movements in these directions and used to evaluate the performance of a candidate design using the objective function in Eq. (3). An evolutionary programming optimization algorithm^34,35^ was used to then generate and identify an optimal design. An initial pool of 1,024 candidate solutions are generated by randomly choosing values for each parameter according to a uniform distribution. Each solution is then evaluated and ranked. The worst solutions are then removed from the pool, while each of the remaining solutions are randomly mutated, according to a normal distribution, to repopulate the solution pool. The variance of this normal distribution decreases with every generation (i.e., simulated annealing) to ensure both initial exploration of the solution space and final refinement of the solution. 32 of the best solutions are maintained in addition to each new generation with no mutation. After 128 generations, the best solution of the final generation is used as the final design. The parameters of this final design were used to position the cable origin points on the frame of the physical device, as well as the cable insertion points on the head piece (Fig. 2, left). Cable length vs. angle data is shown in Fig. 2, right. Note the generally steep slopes indicating a high transmission efficiency across the entirety of each direction of movement.

### Low-level control

The controller tensions each cable by commanding a current *I* to each motor based on the spool radius $\rho$, the motor torque constant $k_t$, and the cable tension $t_i$:

4 $$\begin{aligned} I=\frac{\rho }{k_t}t_i. \end{aligned}$$

#### Tension distribution

Given a desired wrench ${\textbf{w}}$, there exists no analytical solution to determine ${\textbf{t}}$ as *A* is not a square matrix and thus is not invertible. To properly distribute the tensions in each cable to apply a desired ${\textbf{w}}_{des}$ on the head, we formulated the following problem using quadratic programming:

5 $$\begin{aligned} \underset{{\textbf{t}}}{\arg \min }~g({\textbf{t}})=\left( {\textbf{w}}_{des}-A{\textbf{t}}\right) ^\text {T}\left( {\textbf{w}}_{des}-A{\textbf{t}}\right) , \end{aligned}$$

which seeks to minimize the absolute difference between the desired and actual wrench applied by the robot, subject to cable tension minimum and maximum constraints. This objective function has the gradient:

6 $$\begin{aligned} \varvec{\nabla g}_{7\times 1}=-2A^\text {T}\left( {\textbf{w}}_{des}-A{\textbf{t}}\right) , \end{aligned}$$

which can be used in a gradient descent algorithm to solve for the optimal ${\textbf{t}}$, where each cable tension $t_i\in [2,40]$ N. This range was determined experimentally to be able to apply sufficient moments on the head while avoiding deformation of the head piece and slackening of cables. The quadratic objective function ensures the gradient descent algorithm is able to find a global minimum to Eq. (5). Due to the underdetermined nature of this problem, there exists infinitely many solutions for ${\textbf{t}}$. Initializing ${\textbf{t}}$ at its minimum values ensures that the solver will identify a solution that minimizes ${\textbf{t}}$ due to the nature of gradient descent. If no solution exists within the cable tension limits, the solver identifies the closest solution that satisfies the constraints, prioritizing user safety. The solver is allowed to search for 80 ms to ensure the real-time controller (OpenCR1.0, ROBOTIS, Seoul, South Korea) runs at a constant rate $f_c=12.5$ Hz, which provides a good balance between smooth performance and ability to reach a cable tension solution.

#### Forward kinematics

To evaluate *A* at the robot’s current configuration, the positions of the cable insertion points must be known. Given the motor angles, and therefore the lengths of each cable, we use a trilateration algorithm to solve for the positions of these three points in 3D space relative to the global origin. This is made possible by the 2-3-2 cable attachment configuration^28^.

When the position of these three points are known, the position and orientation of the head can then be calculated. The position of the head can be taken as the position of the back insertion point. We describe the orientation of the head $\varvec{\theta }_{3\times 1}$ using the axis-angle representation, where $\varvec{\theta }$ points in the direction of the axis of rotation and $\Vert \varvec{\theta }\Vert$ is equal to the angle of rotation. The components of $\varvec{\theta }$ correspond to flexion-extension, lateral bending, and twist angles. $\varvec{\theta }$ is a zero vector at the neutral upright position of the user. This representation was used because its translation into a moment vector is straightforward, which is crucial for function of the high-level control modes.

#### Device calibration

During calibration of the robot, we establish the user’s neutral position and ensure the controller has accurate knowledge of the length of each cable. First, the cables are unattached from the head piece and the motors are fully retracted to their zero position. Since we know the lengths of the s-hook carabiners as well as the cable loops on the head piece, the controller can determine the lengths of the cables based on motor angles after this step. This is required for forward kinematics. The cables are then attached to the head piece, and the user is asked to sit up and look straight ahead to achieve their upright neutral pose. In this position, the robot calculates the positions of the cable insertion points. $\varvec{\theta }$ can then be calculated relative to the neutral position.

### Benchtop validation

To validate the low-level performance of the robot, we conducted two experiments to verify the forward kinematics and wrench output of the robot. The orientation and wrench accuracy of the robot is summarized in Table 1.

**Figure 3:**
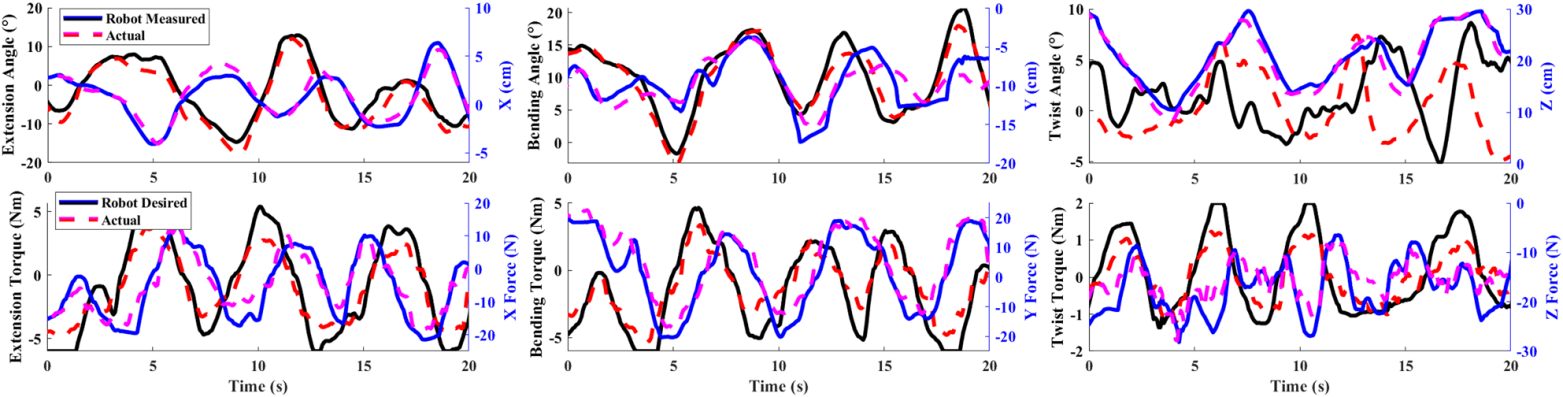
Forward kinematics and wrench output validation plots. Top: Robot measured $\varvec{\theta }$ (solid black), ${\textbf{x}}$ (solid blue) and $\varvec{\theta }_{act}$ (dashed red), ${\textbf{x}}_{act}$ (dashed magenta) vs. time for each DoF. Bottom: Robot desired ${\textbf{w}}$ (solid) and ${\textbf{w}}_{act}$ (dashed) vs. time for each DoF.

To calculate the measured orientation of the head $\varvec{\theta }$ using its forward kinematics algorithm, the robot relies on the measured position of the cable insertion points. We quantified this measurement accuracy against the “actual” orientation $\varvec{\theta }_{act}$ (Fig. 3, top), as measured by a 12-camera motion capture system (Vero, VICON, Oxford, UK). The markers were positioned at the cable insertion points, and $\varvec{\theta }_{act}$ was computed using a similar forward kinematics algorithm. The position of the head ${\textbf{x}}$, ${\textbf{x}}_{act}$ was taken as the 3D position of the back insertion point as measured by the robot and the camera system, respectively. We generated the motion of the head piece by manually moving the head piece within the robot’s workspace.

To apply a desired wrench on the head, the controller must distribute cable tensions appropriately according to Eq. (5) then command motor currents according to Eq. (4). We quantified this wrench tracking ability against ${\textbf{w}}_{act}$ (Fig. 3, bottom), as measured by a 6-axis force/torque sensor (Mini45, ATI Industrial Automation, NC, USA). We fixed the end-effector in a neutral upright pose and controlled the robot to apply a range of forces/moments, equivalent to that which would be present during normal use of this device.

**Table 1:** Robot performance characteristics.

Rotation axis	Flexion-extension (X)	Lateral bending (Y)	Twist (Z)
Mean absolute orientation error (°)	2.1	1.2	4.2
Standard deviation (°)	1.3	0.99	2.8
Test range (°)	−20–12	−3.2–18	−5.0–7.4
Mean absolute moment error (Nm)	1.1	1.1	0.34
Standard deviation (Nm)	0.80	0.76	0.28
Maximum magnitude tested (Nm)	4.7	5.3	1.2

Translation axis	X	Y	Z
Mean absolute position error (cm)	0.78	1.3	1.3
Standard deviation (cm)	0.84	1.0	0.91
Test range (cm)	−6.6–6.3	-15–0.37	8.3–33
Mean absolute force error (N)	3.2	3.1	2.3
Standard deviation (N)	2.4	2.4	1.7
Maximum magnitude tested (N)	20	23	28

### High-level control modes

We implemented multiple high-level control modes, including a resistive force field and two assistive controllers, to demonstrate the potential uses of this robotic device in human subjects. In general, these control modes compute a desired moment ${\textbf{m}}_{des}$ to apply to the head according to a control law and/or user input. As initially implemented for the human study presented in this paper, the force applied to the head is not considered.

#### Force field

**Figure 4:**
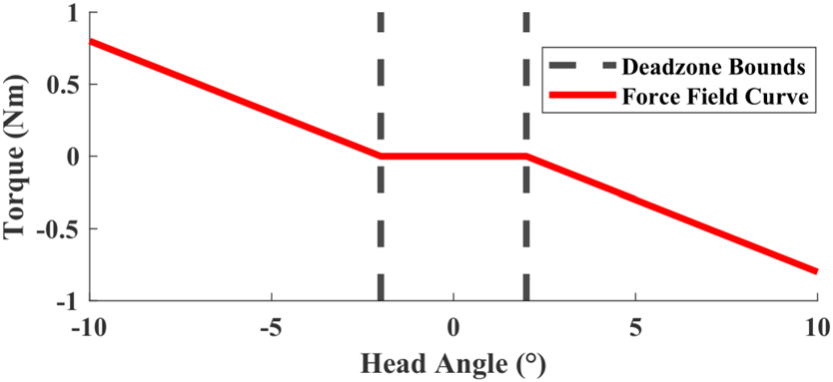
Force field torque vs. head angle for $k_r=0.1~{\text {Nm}}/{\text {deg}}$ and a dead zone $z=2$°.

We created a 3D virtual torsional spring such that the desired moment applied on the head ${\textbf{m}}_{des}$ is proportional to the head angles $\varvec{\theta }$, measured from the upright head pose. In component form, this is expressed as:

7 $$\begin{aligned} m_{des,d}=\left\{ \begin{array}{ll} -k_r\left( \theta _d-z\right) &{}\quad \theta _d>z\\ -k_r\left( \theta _d+z\right) &{}\quad \theta _d<-z\\ 0 &{} -z\le \theta _d\le z \end{array},\right. \end{aligned}$$

where $d=1,2,3$ refers to each axis of rotation, *z* is the dead zone size, and $k_r$ is the force field gain. A sample curve produced by this relationship is shown in Fig. 4. The strength of the force field and the size of the dead zone can be varied by changing the parameters $k_r$ and *z*, respectively. The purpose of this mode is to increase the activation of target neck muscles by increasing the difficulty to perform directional head–neck movements from the upright neutral pose.

#### Torque control

A 3D joystick orientation is mapped to a moment through the following linear relationship:

8 $$\begin{aligned} {\textbf{m}}_{des}=K_m\varvec{\phi }, \end{aligned}$$

where $K_m$ is a $3\times 3$ diagonal matrix of joystick torque gains and $\varvec{\phi }_{3\times 1}$ is the orientation of the joystick. This allows the user to apply a moment on their head to assist with movement. The gains can be adjusted based on user preference.

The torque control mode can be thought of as a “suggestive” assistance mode, as it merely applies a moment on the head. The user is free to resist this applied moment or rely on it fully. When the joystick is in the neutral position, the robot applies zero moment on the head. We also used this control mode to measure the free head–neck movement with the joystick at its neutral position, which controls the robot to apply zero moment on the head.

#### Orientation control

In this mode, the joystick angle $\varvec{\phi }$ maps to a desired head orientation:

9 $$\begin{aligned} \varvec{\theta }_{des}=K_\theta \varvec{\phi }, \end{aligned}$$

where $K_\theta$ is a $3\times 3$ diagonal matrix of joystick angle gains. The controller applies a moment on the head to minimize the magnitude of the error $\tilde{\varvec{\theta }}=\varvec{\theta }_{des}-\varvec{\theta }$ according to the following control law:

10 $$\begin{aligned} {\textbf{m}}_{des}=K_p\tilde{\varvec{\theta }}-K_v\dot{\varvec{\theta }}, \end{aligned}$$

where $K_p$ and $K_v$ are $3\times 3$ diagonal proportional and velocity gain matrices, respectively. A 1° deadzone about the neutral position is used to limit instability. The use of proportional-velocity (PV) control rather than proportional-derivative (PD) control ensures that ${\textbf{m}}_{des}$ does not spike when $\varvec{\theta }_{des}$ changes abruptly.

The orientation control mode is more of a “demanding” assistance mode compared to the “suggestive” torque control mode, as it seeks solely to minimize the orientation error of the user’s head compared to the joystick angle. When the hand is removed from the joystick, the joystick will return to its neutral position due to its internal springs, and the head will be held in the upright pose.

### Safety measures

Safety measures are implemented at different levels to minimize discomfort and risk of injury. Maximum cable tensions are set to 40 N in the tension planner algorithm to limit the maximum moment. Additionally, if any one cable is fully retracted into its cable guide, the power to the robot will be cut. This serves two purposes: i) forward kinematics fails without reliable cable length measurements so behavior may be unpredictable at this point, and ii) this effectively detects cable/carabiner failure and cuts power in that case. S-hook carabiners were chosen to act as mechanical fuses that would release if any one cable reaches too high of a tension. These fail at 70 N, effectively limiting chance for injury should the software limits fail. Finally, a power kill switch is available to immediately turn the device off should any issues not caught by the previous fail-safes arise.

## Human experiments

A study of young, healthy adults was conducted. The goal of this study was to demonstrate the feasibility of this cable-driven device and its proposed control modes in human users. We sought to analyze the biomechanical response of human users while interacting with the various control modes. This study was approved by the Institutional Review Board (IRB #00145893) at the University of Utah. All experiments were performed in accordance with the relevant guidelines and regulations. Informed consent was obtained from all subjects and/or their legal guardian(s).

**Table 2:** Participant characteristics.

$n=12$; 7 Male, 5 Female		
	Mean	StdDev
Age (yrs)	24.1	2.2
Height (cm)	174.4	7.7
Head circumference (cm)	56.4	1.8

**Figure 5:**
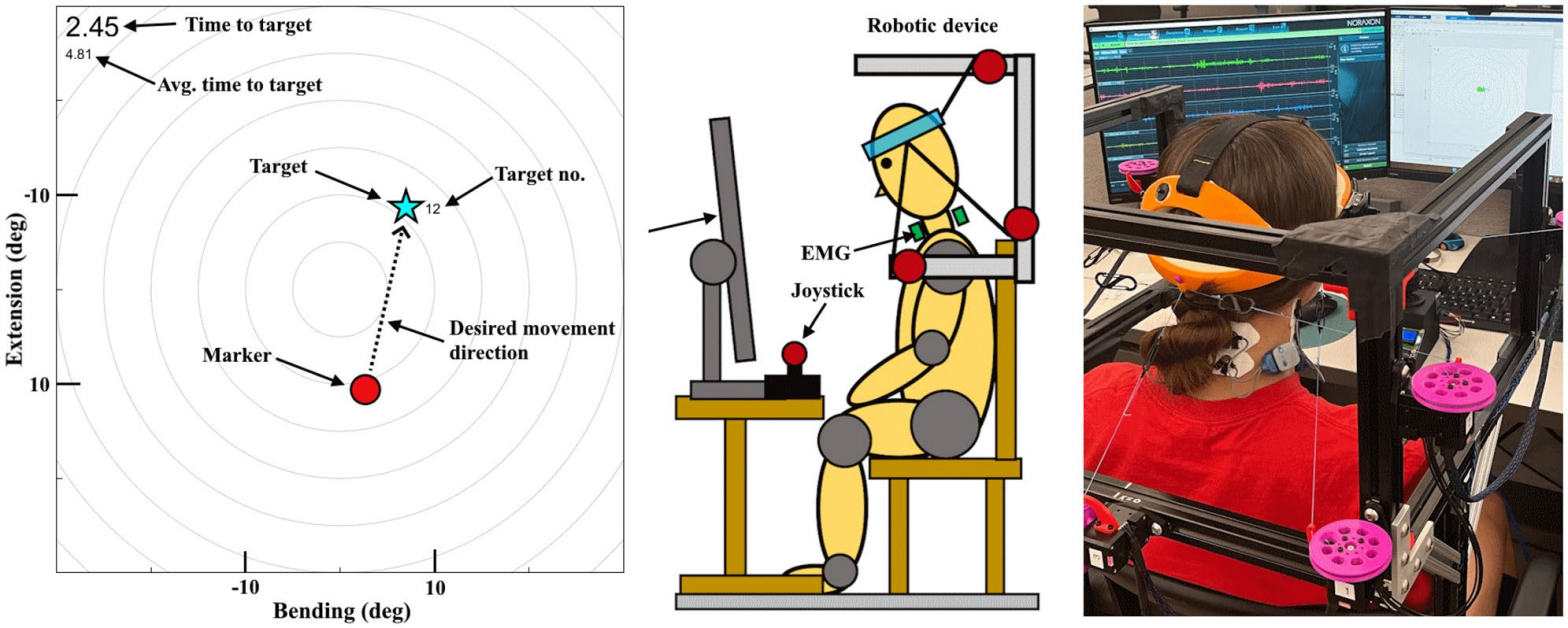
Human study task. Left: Labeled task GUI. The current time to target and average time to target is shown to the participant. The marker color changes from red to green as it approaches the target. The target number counts up to 30 to indicate how far along the participant is in the trial. The flexion-extension axis is flipped so that the GUI represents a top-down view of the head as the participants found that more intuitive. Center: Diagram of experiment setup including (from left to right) monitor, joystick, and chair-mounted robot. Right: Photograph of user study.

### Experimental design

Each participant was asked to reach a visual target with their head rotations while using the robot (Fig. 5). The Cartesian coordinates of the marker (red dot) correspond to the lateral bending and flexion-extension angles of the head from the upright pose, as measured by the forward kinematics model of the robot. The initial position of the target (blue star) was randomly generated, from a uniform random distribution with a random seed, within a square boundary of $\pm 10$° along each axis. This range was chosen experimentally as the task became difficult to see and uncomfortable to perform at greater angles. The target was considered reached when the Cartesian distance between marker and target positions was $<2$°. When the target was reached, the target appeared at a new random position. This was repeated for a total of 30 targets per trial.

A randomized crossover design was used to remove the effect of trial order on the data analysis. Three control modes (force field, orientation control, and torque control) were evaluated by each participant. One controller mode was tested during each trial. For the force field mode, each participant completed a trial for each of two different force field intensities (full vs. half strength). This resulted in a total of 12 trial permutations. We then recruited 12 participants (Table 2) and assigned each to a unique trial order. We also recorded an initial and final baseline trial, before and after testing the control modes, respectively, where zero moment was applied on the head.

While using the assistive controllers, a joystick was given to the participant. The participant was instructed to relax and rely on the robot to move their head as much as possible. For the force field trial, the force field gain $k_r$ was personalized for each participant; a range of $k_r\in \left[ 0.1,0.5\right] {\text {Nm}}/{\text {deg}}$ were tested, and the maximum $k_r$ where the participant was still able to reach $\pm 10$° about each axis was chosen as the force field gain for that participant. This ensures that maximum effort was required from each participant to complete the task. The deadzone $z=2$°. For the half strength force field trial, $k_r$ was reduced by $50\%$. The participant was instructed to use smooth movements but otherwise complete the trials as quickly as possible. The participants were given two practice trials in the zero-moment mode to familiarize themselves with the robot, the task, and the required range of head movement before beginning the experiment.

We measured the head kinematics using the robot and recorded activation of four neck muscles using surface EMG (DTS, Noraxon, AZ, USA). These four muscles are the sternocleidomastoid (SCM) and splenius capitis (SC) on both sides of the body. The kinematics and the EMG were time synchronized. The kinematics were sampled at 12.5 Hz, and the EMG was sampled at 2 kHz. We also recorded the average time to target (ATT) during each trial. At the end of the experiment, participants were asked to give their preference of the two assistive controllers and any additional comments regarding the device and experiment.

### Data analysis

EMG was filtered using a bandpass filter ($f_{pass}=$ 2–500 Hz) to remove noise, followed by full-wave rectification and a normalization to the maximum value recorded during the experiment. We then calculated the percentage increase in mean absolute EMG within each trial for each participant:

11 $$\begin{aligned} \%EMG_{increase}=\frac{EMG_{trial}-EMG_{base}}{EMG_{base}}\times 100, \end{aligned}$$

where $EMG_{trial}$ represents the absolute mean EMG during each trial for each muscle of each participant, and $EMG_{base}$ is the absolute mean EMG during both baseline trials for each muscle of each participant.

We created a visual representation to illustrate the muscle activation pattern with respect to the desired movement direction of the head. For this purpose, we used a moving mean filter (window size of 22°) to smooth the EMG. We then plotted the smoothed EMG against the desired movement direction on a polar coordinate system (Fig. 7, left): (1) the angle of the coordinate is the angular difference between the current marker and target positions, and (2) the amplitude of the coordinate indicates the magnitude of smoothed EMG of the muscle at the same time. For this plot, the smoothed EMG was downsampled to match the sampling frequency of the kinematics data.

For statistical comparisons, the outcome variables were $\%EMG_{increase}$ and *ATT*. The independent variables were the *sex* (male vs. female) and the *control modes* (baseline 1, force field, half strength force field, torque control, orientation control, and baseline 2). Wilcoxon signed-rank tests were used to avoid assumptions about the distributions of the underlying data. When sample sizes were unequal (e.g., when comparing male vs. female results), Mann-Whitney U tests were conducted. The statistical significance level was set at $\alpha =0.05$. Analyses were performed in MATLAB (R2023a, MathWorks, MA, USA).

### Results

**Figure 6:**
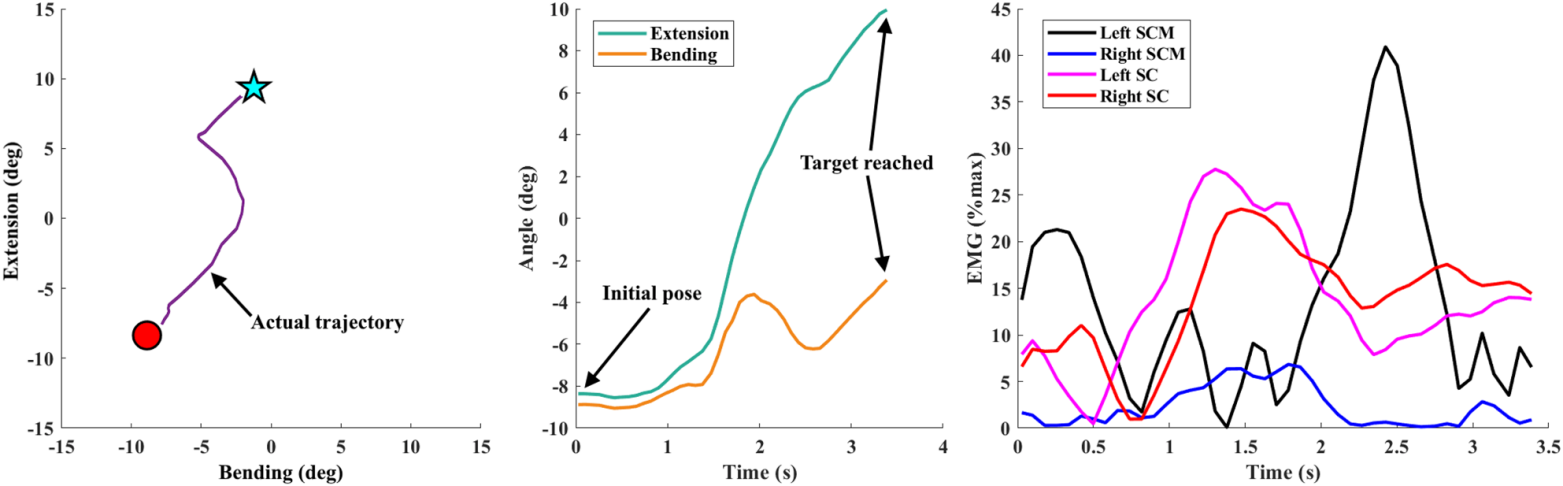
Representative results from one subject reaching a single target. Left: Positions of initial pose (red circle), target pose (blue star), and participant trajectory (purple curve). Center: flexion-extension and lateral bending angle vs. time while reaching target. Right: EMG activity vs. time.

Representative results from one participant reaching a single target during the second baseline trial (zero-moment mode) are shown in Fig. 6. For this single target, the participant started from a combined head flexion of $\sim$ 8° and left bending of $\sim$ 9° and needed to extend the head backward (increase flexion-extension angle) by $\sim$ 18° and bend the head to the right (increase lateral bending angle) by $\sim$ 6° to reach the target. Without any assistive/resistive moment applied by the robot, the head zigzagged toward the target (Fig. 6, left); the flexion-extension motion was mostly smooth but the lateral bending motion was not (Fig. 6, middle). The time to target was measured at $\sim$ 3.4 s. The EMG data (Fig. 6, right) matched the kinematic observations, showing high activation in the SC muscles (responsible for neck extension) and moderate activation in the right SCM muscle (responsible for right bending of the neck) for the task. There is also high activation of the left SCM (responsible for left bending of the neck) observed when the subject moved close to the target, which can be explained by the bending to the opposite direction (at 2–2.5 s) displayed in the lateral bending angle of the head. This demonstrated the coordination of the neck muscles to perform this task that required two head rotations.

**Figure 7:**
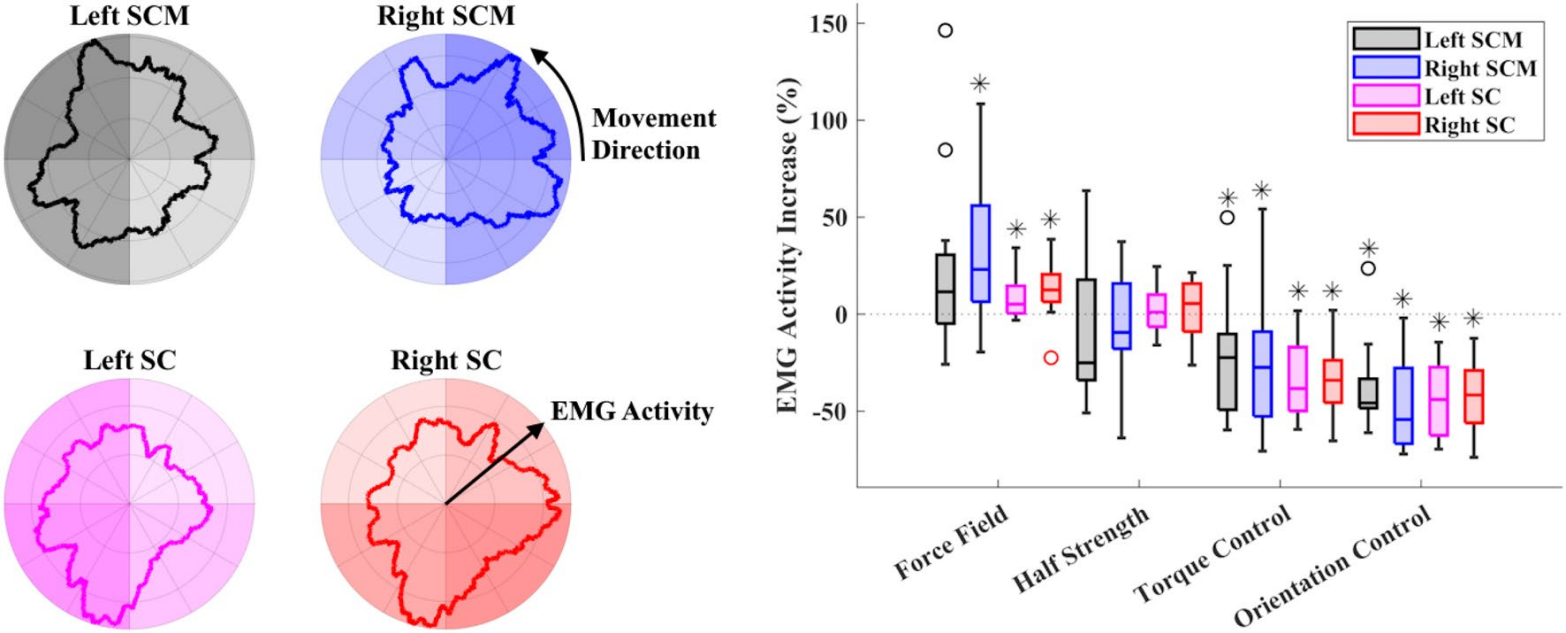
EMG results. Left: Mean EMG vs. required head movement direction (top-down view), plotted on a polar coordinate system, across all participants during the force field trial. Each muscle’s plot is divided into four quadrants; different shading intensities are used to indicate the relative mean of the smoothed EMG in each quadrant. Right: Participants’ mean percentage increase in absolute EMG, grouped by task and then by muscle. Asterisks (*) indicate significant results per muscle.

**Table 3:** Cumulative EMG data.

Task	EMG increase (%)	StdDev (%)
	$*\Leftrightarrow$ Significant results	
Force field	18.8*	32.4
1/2 Strength	− 2.68	24.1
Torque control	− 28.3*	28.1
Orientation control	− 42.9*	21.4

**Figure 8:**
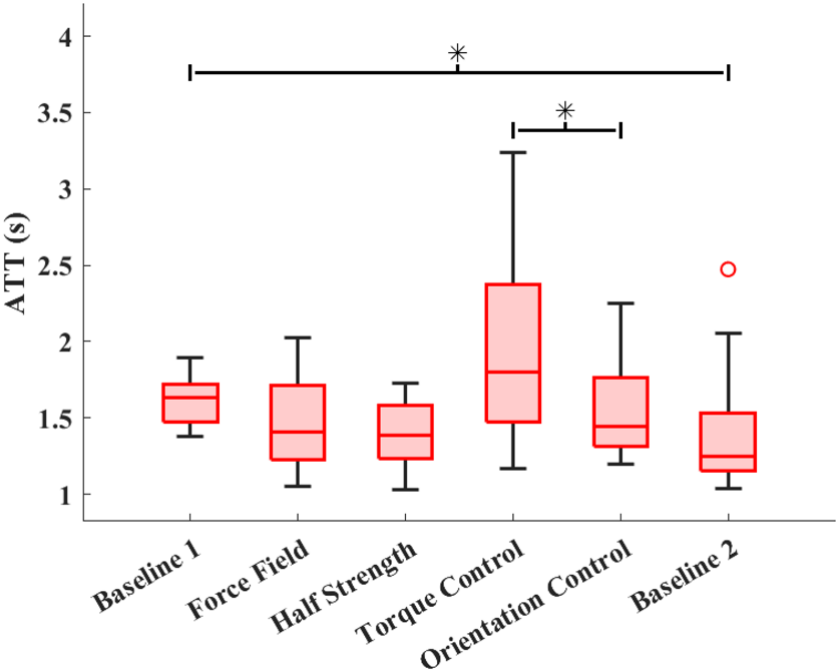
Average Time to Target (ATT) for each trial across all subjects. Asterisks (*) indicate significant differences between trials.

Figure 7, left further illustrates this coordinated muscle behavior in all participants across all conditions. The left SCM muscle activated the most when the task required a combined forward flexion and left lateral bending from the participants’ current head pose, as the EMG intensity is the highest in the second quadrant of the left SCM plot. The activation of this muscle decreased when the target required other combinations of head movements and the least activation occurred when the target required a combination of backward flexion-extension and right lateral bending. Similarly, the right SCM, the left SC, and the right SC muscles activated the most when a combined forward flexion and right lateral bending, a combined backward flexion-extension and left lateral bending, and a combined backward flexion-extension and right lateral bending, respectively, were required. Each muscle activated the least when the required movements were in the opposite quadrant.

Cumulative across all four muscles, the force field mode resulted in a significant ($p<0.001$) increase in mean EMG, and both assistive control modes resulted in significant ($p<0.001$) decreases. The half strength force field had an insignificant ($p=0.55$) effect on EMG magnitude relative to the baseline. The orientation control mode had a significantly ($p<0.001$) greater decrease in EMG than the torque control mode had. These results are summarized in Table 3. Individual muscle data are shown in Fig. 7, right. Additionally, there were no significant ($p>0.12$) differences in EMG increase between male and female participants for any trial.

ATT data for each task are shown in Fig. 8. On average, the torque control mode was completed significantly ($p=0.005$) more slowly than the orientation control mode. The second baseline trial was significantly ($p=0.032$) faster than the first baseline trial. There were no significant ($p>0.76$) differences in ATT between male and female participants for any trial.

## Discussion

The results of the benchtop validation show that the integration of the controller, motors, and cable structure is effective in measuring head position and orientation and applying a desired wrench on the head. To address a shortcoming, these results suggest that orientation measurement about the twist axis is 2–3 times worse than flexion-extension and lateral bending, with a standard deviation $\sim$ 2 times greater. Because of this, we chose to use flexion-extension and lateral bending for the human experiment task. For assistive purposes, improving the twist axis performance is crucial as twist is important for horizontal vision and performing daily activities. Future design optimization of this device may include updated parameters to prioritize performance about the twist axis. Specifically, Eq. (3) could be altered to include non-unit weights for each rotational direction; then, for example, the weight corresponding to the twist axis could be increased to improve twist angle measurement and further improve twist torque production.

In its current chair-mounted form, one application of this robot is to facilitate in-clinic rehabilitation training in CP patients. Using the force field mode, we demonstrated that resistive moments applied on the head in a controlled manner while reaching for visual targets can increase activation of targeted neck muscles. As exercise-based therapies are often used to improve motor functions in CP^13,14^, we envision that this device could be used to develop new paradigms to train head control and maintain horizontal gaze for patients with CP in a quantitative and controlled manner. Differences in percentage increase of EMG activation is similar between sexes, suggesting that sex is not a defining characteristic in the biomechanical response to this control mode.

We hypothesized that using a half strength force field would result in a lower, but still positive, increase in EMG activation compared to full strength. This was not the case; rather, EMG activation during the half strength condition was similar to the baseline trials among all participants. This suggests that the relationship between the force field strength and muscle activation is very nonlinear. Further studies are necessary to determine the optimal resistance level for physical training or to generate a model to achieve desired muscle activation levels. Nonetheless, we demonstrated that the amplitude of the resistive moment can be adjusted to tailor the needs and ability of a patient in a quantifiable and programmable matter.

We also showed that activation of specific neck muscles is associated with the direction of head movement required by the target position relative to the user’s current position. Our results grossly match the observations of activation patterns of neck muscles in healthy individuals during head rotations^18,36^; a forward flexion of the head requires activation of both SCM muscles while a backward flexion-extension requires activation of both SC muscles, and a lateral bending of the head to one side requires activation of the ipsilateral SCM and SC muscles. This validates the ability of the force field mode to apply an appropriate directional resistive moment on the user’s head. It also suggests that this resistive force field control mode could be useful for personalizing therapies; a physical therapist, for example, could define tasks with specific head–neck movement directions based on the targeted muscles of their patient.

We developed two control modes to demonstrate possible means of using a neck robot to assist with head–neck movements. We compared neck muscle activation, task performance (via ATT), and user preference of these two controllers for the target-reaching task with visual feedback. We showed that both assistive control modes can reduce neck muscle activation in healthy participants of both sexes, effectively demonstrating that both modes provided movement assistance during the task. Although participants showed improved task performance (i.e., lower ATT) and higher reduction in muscle activation using the orientation control mode, most preferred the torque control mode. Participants preferred torque control for its smoothness, ease-of-use, and forgiveness to erroneous joystick input. Some participants did in fact prefer orientation control for its precision and predictability. This suggests that further studies are needed to identify optimization objectives in order to personalize assistance for neck robotic devices.

Compared to the first baseline trial, ATT data showed that participants used less time in the second baseline trial to complete the target-reaching task. This may suggest a learning effect of the participants for the task. The ATT during the second baseline trial had a greater variability than the first baseline, which may be attributed to fatigue in some participants. For a true baseline measurement of neck muscle EMG, the robot would ideally apply a net zero moment on the user’s head. However, due to the inherent friction, this was difficult to achieve. Additionally, the effect of optimizing cable placements to maximize transmission ratio results in a robot that is more difficult to backdrive. To improve upon this, lower gear ratios, larger spools, and an adjusted optimization objective function could be utilized.

Currently, this robotic device is chair-mounted. Thus, it cannot yet be used as a wearable device for daily uses. However, because this robot is highly reconfigurable, it provides us with a valuable tool to systematically study design and control for this application. Additionally, the presented optimization can be used, with minimal modifications, to inform design of more compact, suit-like wearable devices. As a demonstration, the robot was controlled to apply a moment on the head. However, as the cable structure enables full 6-DoF control, future research will be conducted to evaluate other control schemes to leverage this ability for rehabilitative purposes. Additionally, other previously mentioned scientific and clinical applications like cervical traction and head perturbation studies will be explored. Users did not report any feelings of constrained or unnatural movement, suggesting great value in the unconstrained nature of the cable-driven design.

## Conclusion

This paper presented the design and control implementations of a novel cable-driven robotic platform for 6-DoF head–neck movements. This robot enables unrestricted head–neck motion and can be accurately controlled to apply a 6-DoF wrench on the head. A study in healthy participants demonstrated this robot’s ability to apply resistive moments on the head to increase target neck muscle activation and assist with head motions through multiple assistive controllers. This robot can be a powerful tool for studying the design and control of cable-driven robots for restoring head–neck movements which has the potential to improve the quality of life in individuals with head–neck mobility limitations, as well as for other scientific and clinical applications.

## Data Availability

The data presented in this paper are available from the corresponding author upon reasonable request.
